# PROMOTING THE LIVED EXPERIENCE OF OLDER ADULTS USING APPLIED QUALITATIVE METHODS: LESSONS LEARNED

**DOI:** 10.1093/geroni/igae098.0947

**Published:** 2024-12-31

**Authors:** Sarah Neller

**Affiliations:** Chair

## Abstract

This symposium brings together five complementary papers focused on promoting the perspectives of older adults using applied methods in qualitative research. It underscores the importance of embedding the lived experiences and unique perspectives of older adults into the fabric of research processes and outcomes. Neller et al. present a six-step process for framing, critically evaluating, and revising prior knowledge within qualitative research. They discuss the development and advancement of a conceptual framework from their study exploring older adults’ experiences creating a legacy of values. Canham et al. present a case example of a community-based participatory knowledge mobilization project across Canada. They report lessons learned from their lived experience advisory board members as they implemented community-engaged initiatives to reduce the stigma experienced by older adults experiencing homelessness. Mahmood et al. discuss digital storytelling as a method of co-creating narratives with older adults experiencing homelessness as a powerful tool to advocate for policy change and address the needs of marginalized older adults. Grittner and Walsh explored the needs of older adults who have experienced homelessness using a trauma-informed design. They present design strategies to foster supportive housing for older adults. Ketcher et al. describe how they developed a qualitative analytic framework to incorporate researcher and community members’ perspectives in the analysis process. This process contributes to the methodological rigor of conducting qualitative analysis with a community-based participatory research design by reflecting community and cultural values. Presenters will emphasize innovative methods and lessons learned when conducting person-centered qualitative research across diverse demographic groups. Qualitative Research Interest Group Sponsored Symposium

